# Fillings

**Published:** 1882-03

**Authors:** J. M. Hurtt

**Affiliations:** Peoria.


AMERICAN JOURNAL

OF

DENTAL SCIENCE.

Vol. XV. THIRD SERIES—MARCH, 1882. No. 11.

### ARTICLE I.

### *Fillings.*

BY DR. J. M. HURTT, OF PEORIA.

[Read before the Illinois State Dental Society.]

Much has been said well and done well in operative dentistry, resulting in many permanent operations. The man who has the ability to follow Doctor Arthur by using that nicety of discrimination in the teeth to be operated on, and then patiently executing the skillfully and delicately polished surfaces required by his theory, demonstrates courage and indomitable energy.

Those operators who are constantly beveling teeth for self-cleansing surfaces, necessarily avoid contour operations. Young operators who produce frequent contour fillings must occasionally fail; and if they do not protect cusps of pulpless teeth, some must be lost. And need we be surprised that from these the " new departure " receives new allies?

Young graduates sometimes risk their reputation on amalgam, and afterwards wish they hadn't. An old practitioner may use cement and gutta-percha with a hope of saving his aching back and enervated nervous system, while theoretically he expects as good fillings as before; but, alas, proves the theory a practical untruth! Therefore, let us cull the principles which, when carried out, result in durable operations, remembering as well that we must, necessarily, occasionally fail.

When gold, amalgam, tin or cement remain in teeth only a few weeks, the retaining grooves are usually at fault. When they remain a year or more, we must look further for the true cause of failure. As we depend largely on the pits, grooves and general anchorages to retain our finished fillings, let us look at their preparation a few minutes. The rubber dam should be applied, when practicable, before commencing to excavate, for there are few cavities which can be prepared properly without its previous careful adjustment. Grooves near the grinding surfaces of a tooth should be strong, and made in a direction to accomodate that finish of the filling which will so antagonize occluding teeth that the force in masticating will be concentrated toward the centre of the organ, rather than distributed to the periphery. Also, suppose it requires two hundred grindings of the teeth at each meal to prepare food for insalivation ; at this rate over two hundred thousand occlusions will be required each year, which suggests the strength requisite in the grooves to resist this force.

Pits or grooves in large cavities in proximal surfaces of incisors and cuspids, if made wholly in the dentine, commencing near the cutting edge, and proceeding toward the pulp, being careful not to approach too near that organ, will retain a filling better, and be more easily filled, than when made in the opposite direction. In this way the point of the tooth is not weakened, and the cutting force of the lower teeth will not be apt to dislodge the filling, if these retainers are well filled. This pit or groove should not be filled with soft foil, but cohesive—a very narrow, short strip of, say No. 20, or No. 30 is best, filling very carefully, and over-lapping the edges, in order to weld the gold in the retainer, by bands, to the body of the filling.

A small gold screw, if driven into the dentine at this point, is still better, allowing the screw to cut its own thread. This is a difficult operation, for, if the sides of the screw touch the enamel plates, they will be checked, and, after filling, either discoloration or decay ensue. In large cavities in bicuspids and molars, the retainers should be made in the dentine, if possible, using the enamel to assist in retaining the filling when it is very resistant, and only then to assist in forming a general dovetail. There are few exceptions to this rule. When making general anchorages in grinding surfaces, remember that all cusps in pulpless teeth are to be protected from being split off. Retaining pits in the cervical portion of a cavity should be wholly in the dentine.

Fillings are lost from cavities prepared by good operators, for man in his entirety is imperfect, and his labor, partaking of this bias must bear the imprint. Also the best patrons do not carry out strictly our simplest instructions for the preservation of their teeth. With this in mind, we may examine the reasons for fillings being lost.

Tin fillings gradually wear away except on unused surfaces, and usually leave the walls uninjured, if the cavities are properly prepared and carefully filled. Gutta-percha is worn away as much as tin, but it does not preserve the walls as well, for sometimes we see extensive decay at the cervical margins of these fillings. Similar decay occurs around cement fillings, when we have been careful to avoid it, and even in those teeth that are said to indicate its use, chalky and pulpless ones. These cavities are due either to minute air vesicles at the margins, improperly mixing, improper proportions, slowness in inserting the filling, or not keeping the filling dry long enough. In cavities of chalky teeth, in surfaces cleansed in masticating, and in proximal where they do not extend to the gingival border, cement is the best filling until a change of the tooth structure takes place. In healthy mouths where nothing is eaten between meals, we may succeed with cement in cavities extending under the gum. Decay may occur anywhere around an amalgam filling, or the amalgam may disappoint by preserving the tooth. In my observations these are rules and not exceptions. A good cement wears away less rapidly than tin, and often leaves the walls in good condition, while amalgam does not wear away except at the margins. Cement is easy of insertion, and disappoints by cavities forming under the gum, nearly exposing the pulp, while the surface seen looks well. Amalgam may have decaying dentine under its entire surface, and the pulp slowly perishing, while its external surface looks bright, and nothing but a mere line of demarkation appear as a sign of this destruction below.

These defects are rarely noticeable in gold fillings, bearing in mind painstaking, patiently-executed operations. Gutta-percha in all its forms, when used as a filling, is but a temporary stopping in my hands.

Amalgam fillings fail from expansion, contraction, improper insertion, improper finish and oxidation; and no matter how carefully instructed, or diligent to learn the principles governing these, the operator is just as liable to failure where he needs the effect of his training as he is to have a better filling where he is indifferent as to the result; and the novice is at still greater disadvantage.

Gold fillings fail from improper preparation of the cavities, want of adaptation to the walls, marring the margins in consolidation, roughening the edges and surface of the tooth around the filling when finishing, with a failure to properly polish this surface, especially at the cervix; allowing softened portions of enamel to remain because difficult of access and sensitive to the excavator, risking a failure rather than a temporarily disagreeable impression; an improper finish of the occluding surface; the insertion of a gold filling where it is contra-indicated; too severe malleting; wedging a piece of annealed gold between a wall and the main body of filling, to repair a defect after the gold is consolidated, causing undue pressure.

All the causes herein enumerated may be modified and frequently avoided by careful manipulation and studious attention to details. I am aware, however, that there are many operators whose conceit and inaptitude in manipulative ability preclude the possibility of success in the finer operations of operative dentistry; or, perhaps, better say the converse of diligence prevents success.

I am aware, too, that there are many sensitive natures who have the keenness of foresight, pliability for adaptation, latent skill and tact, sense of honor and ambition, to bless us by their advice and society, but who are too modest to attempt any public display for fear of criticism, and cannot openly canvass the operations, essays and conduct of those who care less for such judgment. We should encourage these by the hand of fellowship, enabling them to stem the tide and make an effort, despite forebodings and much perspiring. But beside all this, we owe it to our society and fellow-men beyond this narrower circle, to give whatever data we think we have, though humble, in broken sentences, and sometimes in imperfect clinics.

I have at times found men who expressed in the finest dicta, the principles of practical operations, almost totally incompetent to carry them out. On the contrary, I have found men who would tremble and alternately perspire and chill while in their seats, at the thought of attempting to speak in open society, and who, when they did speak, uttered broken sentences, saying almost totally the opposite of what they intended, becoming disgusted with themselves for making the attempt at all, not only capable of carrying out the principles enunciated by others, but were themselves astonished to find that they were really good operators. Some of the best fillings I have seen, and these of gold, were from the hands of men who had passed through the trying ordeal of learning to talk in open society. Every member should be encouraged to emulate those who have learned to speak well in public.

As gold has been attacked many times during the past few years, and the cheaper fillings bolstered by many a pen, let us see if there are not some redeeming features about practical reasons for our failures with gold. Some of these have been urged upon your attention and been disregarded. One of these is the improper preparation of the cavity. The literature of this society is replete with information on this subject; yet I wish to again say a few words here. I repeat, it seems to me impossible to properly prepare a cavity without first adjusting the rubber dam. Excavating is less painful after the cavity is dry, the walls and recesses are more readily disclosed, and in many cases the supposition of a pulp exposure is dispelled. I am satisfied that the truth has not been sufficiently impressed on our minds that the cervix should be excavated until the margin is smooth, solid, and free from a prominent ridge. There are softened lines of enamel near the cervix, which extended laterally from cavities in proximal surfaces, which demand removal for success. Opportunities for seeing operations at the hands of another should not be lost if we would have points of inestimable value discover themselves to us. In the clinics, follow the operator from beginning to end, and thus prove a patient student. In very large cavities, fill the main portion with cement, and while pliable, make, with a blunt excavator or old plugger, the size you wish, retaining pits in the cement, and after hardening remove any protruding surplus obstructing the view, and partially depend on these to anchor the fillings. In many cases these retainers will be sufficient.

Many dentists commence filling by introducing pieces of gold too large for perfect adaptation to the walls. The edges should be true and smooth, but if a larger pellet is placed against the wall than can be made solid, an attempt will be made to condense it by undue pressure towards the face of the tooth, and thus crush the margin, inviting certain decay; or, if the gold is not condensed against the floor or wall, a defect will remain after finishing, that will cause future trouble. It frequently happens that a larger piece of gold than is suitable is forced into a groove or pit, and the wall fractured or broken, and the tooth afterwards decays at that point. Another, and one of the most prolific causes of failure of gold fillings is the finishing or want of true finish to the fillings and the enamel surface. In filing, great care should be taken not to mar the tooth, and to avoid a possible roughening of enamel about the filling.

A thorough polish is of great benefit; one minute is not enough—five, ten, twenty, an hour—according to circumstances. You will become weary, and then your patient sometimes complains; be faithful and honest, and if you have been kind he will come to you again with cheer. I believe there is more in the preservative effect in those fillings which are water-tight, from leaving the enamel surface untouched, than from all the chemical and electro-chemical theories heard of. That the margins of the tooth around gold fillings in proximal cavities can be finished without marring the polished enamel, I know. But no careless, impatient dentist, who cares for the gain only, is capable of learning how it is done. Gold is the best material yet found for the *great majority* of cases, and no other filling material now known will save them equally well. As the expert must learn how to finish a filling of gold, so the bungler must rise to an immensely higher plane to be able to properly fill a cavity with gold.

Fillings that are too full and approach too near the pulp, will, in the close approximation to that organ, by continued strokes, eventually cause inflammation, and a deposit of "calcific granules" in its substance, and, assisted by thermal changes result in its devitalization.

When the filling is too full at the grinding surface we have the condition of an undue closure against it, and, unless the unfilled organ occluding the one filled, is worn away twice the amount of those without fillings, it will act as a constant source of neuralgia, and a continual reminder of the one-fourth million necessitous occlusions to keep up a year's mortal existence. The effect of the general wearing away of the teeth is not a release of the pressure on fillings, because metals, such as gold and amalgam, are hardened on their hammered surfaces, and the oftener they are occluded the harder the surfaces become, until in the case of malleable amalgam and gold, the filling must break asunder, the walls of the tooth yield, or the tooth take on inflammation. Therefore, gold and amalgam fillings should be dressed off when they occlude an antagonist, at least once a year. Under this constant physico-dental malleting the gold expands laterally, and sometimes causes pressure, resulting in dentine enamel disintegration. If this pressure is not removed so that the teeth will close evenly, the pericemental membrane will become thickened, and in the case of a tooth with a live pulp, produce a "calcified deposit" in that organ or upon the root, or in the case of a pulpless tooth, take on inflammation, which, becoming chronic, will result in a fistula, with constant flow of pus.

DISCUSSION.

Dr. Townsend.—There are two points in Dr. Hurtt's essay to which I desire to call attention. One is the chalky enamel, which is often left next the lateral walls of proximal cavities in molars and bicuspids. I have no doubt that many failures are caused in that way. In most cases there is a softened condition of the enamel extending laterally on each side of these cavities; the surface is smooth, and the exploring instrument passes over it so smoothly that the operator is often deceived. The color, which is lighter and more opaque than sound enamel, will alone enable us to distinguish it. After the surface is penetrated it is found more or less softened.

Where I cut through from the grinding surface I cut away the lateral walls sufficiently to include this softened portion. The advantages of this method are many. The unsound enamel is all removed. The gold in the adjacent teeth comes in contact, and not the enamel. Any food allowed to remain between the teeth lodges against the filling instead of the enamel. The lateral margins of the cavity are in plain sight, and easily examined; and last, but by no means least, the lateral margins of the filling are easily cleansed with the brush.

The other point I wish to speak of is retaining points near the cutting edge in proximal cavities in incisors. The best means I have ever found for retaining fillings in these cases, where the corner is weak or gone entirely, is a retaining screw, inserted diagonally, starting in as near the cutting edge as possible without touching the plates of enamel, and drilling in a direction from the point of the tooth somewhat toward the gum on the opposite side instead of at a right angle, care being taken not to encroach upon the pulp. In thick teeth with small pulps this is easily accomplished; but in thin teeth with large pulps it is extremely difficult to insert a retaining screw successfully. If it touches either plate of enamel it will fracture it, and to start sufficiently high up to avoid the enamel endangers the pulp; but the advantage to be gained in the increased strength afforded to a filling of this kind by a well inserted retaining screw are such that I feel justified in running some risks to secure this end.

Dr. Talbott.—Do you always cut through from the grinding surface in molars and bicuspids?

Dr. Townsend.—I do in the majority of cases, but not in all.

Dr. Noyes.—The arch of enamel (and dentine) at the proximal corner of a bicuspid is its principal strength to prevent the splitting of the tooth or the breaking off of one of the cusps under the force of mastication, and I do not think it justifiable to spoil that arch by cutting from the crown merely for the sake of an opportunity to work. I would spend a week in wedging such teeth apart sooner than notch the grinding surface unnecessarily. If the cavities are so large that the overhang is composed wholly of enamel, of course it had best be cut away.

Dr. Taylor.—One's practice is modified by his surroundings. Dr. Townsend and myself often have patients from the country who may not be able to come again for a month or two, and we cannot have the four days or a week in which to wedge the teeth apart, but must do the best we can. Dr. Miles' paper referred to the time when it was common for a dentist to have several other callings. That day is not quite yet gone by. There is a dentist in the town where I live who is also a music teacher, a photographer, a healer of piles, etc., etc.

Dr. Townsend.—I never cut through from the grinding surface merely to gain room for operating, but because I believe that I can better preserve the teeth in a majority of cases in that way. I wedge apart until sufficient space is gained to finish the filling after the contour is fully restored, and often instruct country patients to wedge the teeth themselves before coming. In soft teeth, with a tendency to proximal decay, and especially in young persons, I almost invariably cut through, but in hard, dense teeth of older people it is often not so necessary.

Dr. K. B. Davis.—There is not much difficult in making good operations in grinding surface cavities, but proximal cavities in bicuspids and molars are the difficult cases. A very prominent error is often seen in the way such fillings are finished. In natural jaws of the best models we see but one point of the proximal surface of a tooth in contact with its fellow. Suppose there are cavities in both teeth extending to the gum; they are often filled and finished so that they touch from crown to cervix, having far larger surfaces in contact than before. If we study nature's models we shall restore the original shape, when they will be safer than at first, because the most exposed points are now gold. They should be finished, if possible, so that two convex surfaces of gold will touch.

Dr. Hanaford.—Do you bevel the lateral margins of such cavities?

Dr. Davis.—A very little, not enough to make an attenuated edge of gold.

Dr. Harlan.—I think the way to properly finish such fillings is to use the thinnest separating files to separate the fillings, and finish at the neck with strips of emery paper, making the teeth smaller at the necks, so that the natural shape is retained, and they are easily cleaned at the margin of the gum.

Dr. Talbot.—In a crowded arch we sometimes see a first molar much decayed, and the convexity of the adjoining bicuspid crowded into the cavity. In such cases I would separate well for several days, and would cut away the inner corner of the molar quite freely, partly because the small amount of enamel at the neck of a molar is liable to chip off if not removed, but chiefly to increase the space between the teeth at their necks on the lingual side, and leave a space and surfaces easily cleaned.

Dr. Taylor.—I have found the ribbon saws superior to the separating files for cutting through narrow spaces, because they cut only at the edge, and are flexible.

Dr. Black.—(Showed a carrier which he had made for thin files.) It is strong enough to hold them rigidly, so that a thin file may be used with some force in the same way as a heavier one, without breaking. This is important in the finishing of approximal cavities when it is desirable that the teeth be not separated by wedging or otherwise.

In excavating approximal cavities it is very important that a square shoulder be obtained at the cervical margin. In these decays the cavity just within the enamel often extends below the outer margin, leaving some enamel which appears sound ; the gum is much in the way if this be cut away so as to make this portion of the cavity square ; but if it be cut away it is very likely to break in packing the gold, and being so much hidden, may escape notice and work the ruin of the filling. Pits for anchorage are best, placed at the outer and inner angles, so that the cervical wall be not weakened. The gold should not, as a rule, be laid out over the cervical wall at once, but it should be approached from the deeper part of the cavity and gradually built out over the margin, otherwise it is likely to spring off from the margin, causing a leak. To prevent this, we build up to the margin, keeping the condensed gold as thick as the case will allow, and give us a certainty of getting at the margin perfectly. We ought also to condense the cervical portion of the filling, if possible, after the gold is all in. I have here a set of double curved instruments for that purpose, with which I can condense the cervical portion of any filling. Those for the distal cavities have the mallet end curved in a half circle, so that it may be struck in a direction away from the patient. The instrument is twelve inches long.

Dr. Templeton.—What do you think of using tin at the cervical margin for the sake of its therapeutic effect upon the wall of the cavity?

Dr. Black.—I do not think anything of it. If fearful of injuring the margin of the cavity I would use ammoniated foil, which is as soft as any tin. The extra cohesive foil (which condition implies the greatest softness, purity and cleanliness) will become perfectly non-cohesive by a few minutes' exposure to ammonia, without in the least impairing its softness. It may easily be ammoniated by placing in a box or drawer together with a bit of spunk or cotton damped with spirits of ammonia and closed for a short time. It may be kept thus in a special box for use. It will become perfectly non-cohesive, so that it will not weld any more than tissue paper will weld, and makes the best cushion for margins that I have yet tried. I place such thickness as I may desire on my margin and then secure it by placing cohesive gold over it exactly as is done in covering the margins with tin. Whenever we have foil that is not cohesive after annealing, it is because there is something on its surface that heat will not drive off. Phosphorus and sulphur, and perhaps many other things, will accomplish that. Ammoniated foil is easily reannealed, and then welds perfectly. Many of the things that are liable to make foil permanently non-cohesive are of an acid nature and the foil is protected from them by ammonia. All the metals are welding metals if clean. Gold does not attract oxygen to its surface and so is easily welded cold. Platina does attract oxygen and cannot be welded cold, but by covering platina with gold we can get the welding property of the gold and also of the platina beneath it, and they will become a coherent mass under the plugger. Some persons spoil their gold by annealing in a lamp after touching the wick with the phosphorus or sulphur of a match.

Dr. Brophy asked if the saw-frame shown by Dr. Black is manufactured by any one for sale.

Dr. Black.—It is not. I have urged several dealers in dental goods to make them, but have finally had to make one myself for my own use.

Dr. Templeton, of Pittsburg.—I have seen many failures of approximal fillings because not well condensed on the side of the cavity next to the operator. It is often impossible to get the point of a mallet plugger into the bottom of the undercuts in such places so as to be effective, and I am convinced that the best course to pursue is to use soft foil and hand pressure in such places. One thing about this meeting has pleased me very much. I have not once heard the phrase "electro-magnetic mallet." I have become very much disgusted with the enthusiastic devotion of many who use that instrument, who talk of little else than electric mallet, and talk of that about all the time. I was one year on the executive committee of the Pennsylvania State Society, and tried very hard to make a programme without it, but met with only partial success. My objection to the electro-magnetic mallet is this: We all know that a tooth will move a little in its socket under a blow, and rebound or recover its former position after a slight pressure or stroke, and it willl endure it and not get sore if the blows do not come so fast as to prevent its returning to its natural position in the interval between them; but if the blows are repeated too frequently, the tooth is kept vibrating, and I do not believe that gold can be welded on a vibrating surface. There are other objections, in the irregularity and uncertainty of batteries, the difficulty and inconvenience of managing them, and the complicated mechanism of the mallet, the frequent cutting away of much sound tooth structure for the sake of getting the force in a direct line, the cutting or grinding up of the walls of the cavity, rendering it impossible to make a moisture-tight joint, etc. I believe that the best results are obtained by a combination of hand and mallet force.

Dr. French, of Pittsburg.—I am sorry that my friend, Dr. Templeton, should have brought up the subject of the electro-magnetic mallet here, where nothing would have been heard about it but for him. I am afraid that opposition to it is as much of a hobby with him as the defence of it is with some others of our Pennsylvania dentists. The paper said that the rubber dam should be applied for excavating. I believe the dam should be on before any work is done. I have taken pleasure in carefully and thoroughly trying both ways and noticing the different conditions and results, and there is no room to question the very great advantages of having the work dry from the first. Teeth are far less sensitive when dry.

Dr. Morrison.—The more simple the instrument we use the better, if they are adapted to the work to be done. If we go on twenty-five years as we have in the past, no office will be large enough to hold all the appliances and machinery of a complete outfit.

Dr. Brophy illustrated on the blackboard some instruments made by Dr. Talbot for shaping the cervical margins and side walls of cavities, and also illustrated a bicuspid having a hollow proximal face at the neck on which a file will either leave some over-lapping gold or else shoulder the neck of the tooth. Fillings in such teeth must be trimmed at the cervical margin with cutting instruments.

